# The Awareness of the Fascial System

**DOI:** 10.7759/cureus.3397

**Published:** 2018-10-01

**Authors:** Bruno Bordoni, Marta Simonelli

**Affiliations:** 1 Cardiology, Foundation Don Carlo Gnocchi / (IRCCS) Institute of Hospitalization and Care, Milano, ITA; 2 Osteopathy, (SOFI) School of French-Italian Osteopathy, Pisa, ITA

**Keywords:** fascia, fascial system, myofascial, quantum physics, physiology

## Abstract

Fascia is a cacophony of functions and information, a completely adaptable entropy complex. The fascial system has a solid and a liquid component, acting in a perfect symbiotic synchrony. Each cell communicates with the other cells by sending and receiving signals; this concept is a part of quantum physics and it is known as quantum entanglement: a physical system cannot be described individually, but only as a juxtaposition of multiple systems, where the measurement of a quantity determines the value for other systems. Fascial continuum serves as a target for different manual approaches, such as physiotherapy, osteopathy and chiropractic. Cellular behaviour and the inclusion of quantum physics background are hardly being considered to find out what happens between the operator and the patient during a manual physical contact. The article examines these topics. According to the authors' knowledge, this is the first scientific text to offer manual operators’ new perspectives to understand what happens during palpatory contact. A fascial cell has not only memory but also the awareness of the mechanometabolic information it feels, and it has the anticipatory predisposition in preparing itself for alteration of its natural environment.

## Introduction and background

The fascial system supports the human body in its vital functions: it ensures the maintenance of posture and motor expression and helps achieve a salutogenic homeostasis [1-5]. Fascia also influences the emotional sphere [6]. In a previous study, we showed that the fascia not only functions in support and communication, protection and sustenance but also provides protection to the entire body through the epidermis that is an inherent part of the fascia [7]. We provide a new definition of the fascia: “The fascia is any tissue that contains features capable of responding to mechanical stimuli. The fascial continuum is the result of the evolution of the perfect synergy among different tissues, capable of supporting, dividing, penetrating and connecting all the districts of the body, from the epidermis to the bone, involving all the functions and organic structures. The continuum constantly transmits and receives mechanometabolic information that can influence the shape and function of the entire body. These afferent/efferent impulses come from the fascia and the tissues that are not considered as part of the fascia in a bi-univocal mode [7].” Fascia consists partly of solid matter (bones, fat, muscles, ligaments and reciprocal tension membrane) and partly of liquid fascia (blood and lymph), combined in a single functional continuum [8]. Based on the idea of liquid and solid fascia, we have recently presented a new theoretical model, with the aim of explaining the importance of liquids (pressure, direction and velocity) in a final and functional expression of the fascial system: Rapid Adaptability of Internal Network (RAIN) [8]. The fascial system cannot be divided into layers because of its entropic nature based on the highest capacity to adapt itself to different stress scenarios; fascia has the freedom to respond to any stimuli (internal/external), thanks to the lack of predefined structural and fluidic patterns or negentropic behaviour [9]. The nervous system does not regulate the morphological features of the fascial system. The latter is a holobiont, an asymptotic behaviour between the mechanical environment inside and outside the cell and the modification of the environment itself. A non-movement syntropic is based on a heuristic basis: the maximum configuration of the order and at the same time maximum differentiation with the aim to have access to all information [10-12]. Tissues use a stigmergic communication through a stochastic process to achieve optimal adaptation strategies; tissues change their characteristics and the means of transmission of external information inward. It is not only about a tissue, but it is, in fact, an awareness [10,12-15]. The article discusses the fascial cellular response modality to mechanical stimuli and the possible influence on the fascial tissue by a manual palpation during a manual treatment, in terms of quantum physics and physiology.

## Review

Palpation

Palpation is a mechanical induction (with perpendicular or tangential pressure) towards a static (solid) and hydrostatic (liquid) tissue, within a specific period. Palpation is an important part of the physical examination, a manual exploration of the tactile perception [16]. The tactile perception system gathers information about the environment using mechanoreceptors and thermoreceptors residing in the skin, as well as from the deeper mechanoreceptors located in the myofascial and articular system [16]. The palm of the hand has specific receptors, which permit to determine the size of the palpated tissues (Meissner corpuscle and Merkel cell complex) and understand the tissues' ability to deform under a rapid or continuous touch (Ruffini and Pacinian corpuscles). Touch can discriminate a solid feature for about 200 microns [16]. Furthermore, thermoreceptors are capable of detecting temperature variations (myelinated type Aδ fibres and non-myelinated type C fibres) [16-17]. According to the Bayesian perceptive interference, mechanoreceptors and thermoreceptors can detect wetness of the palpated tissues through a multimodal integration [17]. Inspection and palpation activate the superior and inferior parietal lobules in the operator's cortex [16]. Palpation is part of a personal experiential memory bank useful to find tissue anomalies, and it is a manual art [18]. As with all arts, the result is not always reproducible in the same way. In literature, there seems to be some disagreement in the palpatory results between different operators using the same patients; even the palpatory practical experience does not seem to make the difference in deciphering patients' tissue abnormalities [19]. The first tissue touched by hands is the epidermis. If the pressure increases, the soft tissues perceive the tension created, for example, the muscles and the visceral fascia tissues that connect or cover all organs in the body. Fascia is an interrelation of liquids and solids [8]. In physics, the “state of rest” is the macroscopic condition of a body that is not subject to motion [20]. The static (solid objects) and the hydrostatics (fluids objects) study bodies in a state of rest. In case of a fluid at rest, the individual constituent particles (atoms and molecules) move because of the phenomenon of thermal agitation (absent at absolute zero temperature); hence, macroscopically, the fluid is at rest, but microscopically, the individual particles keep moving. Similarly, in a solid body, the individual constituent particles (atoms, ions and molecules) are constantly in motion; however, this movement is less evident than in fluid bodies. While particles in fluids move freely within a volume containing them, this motion of particles in a solid is more like a vibrational motion, i.e., in the case of an ionic crystalline solid, ions undergo minimum translations around their reticular position [20-22]. The pressure resulting from palpation and received by the tissues creates numerous vectors that are dispersed in multiple directions, above ground and in depth [23]. Different models try to explain what happens to a tissue deformed by mechanical stress, but none with a satisfactory solution [24]. What we know for sure is that palpation and manual approaches to tissues can alter the cellular behaviour of the fascial system [25].

Changes in Cell Behavior in Case of Mechanical Stress

Fascial cells derive from tissues developed from the mesoderm and partly from the neural crests of the ectoderm (neck and face): skin, fat, blood and lymph, connective tissues and muscles, other tissues that cover and sustain the nervous, blood and lymphatic system, as well as bone tissues. A seamless web of connective tissues that covers, supports and penetrates the viscera is part of the fascial system [2,7]. Although the difference between the cells in different tissues is evident, their behaviour in case of mechanical stress is very similar. Cellular deformation makes the cell aware of what happens inside it and in the environment in which it lives, resulting in behaviours that can anticipate the deformation [26]. The shape of a cell is a stochastic process based on a perfect relationship between entropy and syntropy, and the second law of thermodynamics is not violated. Every living system cannot die of thermal death; it is in response to the entropy that the system has an opposite syntrophic process of natural restoration of order. An example is the metabolism of living organisms in which anabolism is present in response to catabolism. In living beings, syntropy would act as a tendency to project itself into the future, being a feature coming from the future towards the past [26]. This means that the exploration of the surrounding environment allows us to improve and not repeat the same mistakes: to understand what will happen, thanks to the accumulated experiences. A reasonable attempt to craft a logical explanation for that feature is a part of various studies on pre-stimulus response found in human beings and animals. They discovered that both heart rate and skin conductance and other biological parameters vary before emotional phenomena appear. This would demonstrate the man's instinctive tendency to prevent future acts according to the principle of syntropy [26]. Mechanical events suffered by the fascial holobiont are actively maintained in its memory, with the aim of being already predisposed to a new action of the same stressors [27]. A fascial cell has not only memory, but also has the awareness of the mechanometabolic information it feels, and it has the anticipatory predisposition in preparing itself for alteration of its natural intra- and extracellular milieu. A cellular genome can monitor itself in response to mechano-metabolic stimuli, obtaining information not only from the extracellular matrix (ECM) but also from other cells and tissues. Ribonucleic acid (RNA) is not only a carrier of proteins but also has a great influence on epigenetics. Micro RNAs and other non-coding RNAs (ncRNA, endo-siRNAs, piRNAs, antisense and long ncRNAs) determine cell-to-cell gene activation and expression. Particularly, RNA interference (RNAi) is involved in learning, self-propagation and amplification of information, involving different tissues [28]. Deoxyribonucleic acid (DNA) is involved in transmitting and transporting information outside the cells in different and distant sites from its original site of replication. Circulating ring-like non-genomic DNA seems to be involved in other cells specialization and the production of specific proteins such as titin in cardiac muscles [29]. A cell communicates with the other cells by sending and receiving signals; this concept is part of quantum physics and it is known as quantum entanglement: a physical system cannot be described individually, but only as a juxtaposition of multiple systems, and the measurement of a quantity determines the value for other systems [30]. According to the quantum theory, each element has a non-hierarchical form of organization, and it only responds when necessary (mechanical and metabolic stimulation) [31].

Cell membrane

Nearly all the cell membranes have an action potential, that is, an electric potential difference (voltage) between the cytosol, the interior of the cell that has a negative voltage and the extracellular space that has positive charges. The resting membrane potential is determined by the uneven distribution of the phospholipids and because of this potential difference across the cell membrane, the membrane is said to be polarized. A typical voltage across a cell membrane is between -60 mV and -70 mV [32-34]. This time-varying electric field becomes the source of magnetic and electromagnetic fields. The study of electromagnetic fields in living cells is placed under the aegis of magnetobiology [35]. Any change in the electromagnetic field can deform a cell for a very short time; this deformation improves and accelerates adaptation processes and cellular functions, such as the synthesis of adenosine triphosphate (ATP) or controlling the enzymatic processes of DNA [35]. Cell deformation and the electromagnetic field are influenced and facilitated by the anisotropic rotation of the phospholipids and water molecules sited in the membrane [36-37]. The ions exchanged during an action potential make a change in the cell volume, causing a cell deformation and inducing a transient electromagnetic field. This mechanism creates microwaves, which radiate to other cell membranes influencing the rotation or the orientation of electrons and affecting other electromagnetic fields [35]. We could compare this phenomenon, already decoded for the central nervous system, to what happens to neurons. Whenever an electrical impulse runs along a neuron, a small electric field surrounds that cell. The sum of all the electric fields created by the neural activity modifies the activation of the single neurons, increasing the synchronism of the neural activity. The effect of an increased neural coordination is defined as an ephaptic effect or ephaptic coupling [38]. All cells present in the liquid and solid fascial system have electromagnetic fields stimulated by membrane deformation; the greater the synchronicity of this event among multiple cells, the higher the quantum coherence and the functional cellular effectiveness. This quantum synchronicity in physics is called the Larmor precession [39]. The electromagnetic fields can travel faster than the electrical signal, and they can cross the whole body almost instantly [39]. The cells that make up the tissues show a greater capacity for awareness if they act together [5,12]. The cell membrane has unstable areas, which allow mechanical information to spread widely and to be different. These fewer complex areas or system of pervasive information fields (PIFs) are the cellular entropic border, where PIFs represent the cellular negentropy (also called syntropy), in a perfect balance to keep the homeostasis maintained [26]. Negentropy allows information to be less fragmented and reach the internal part of a cell more effectively, optimizing the entropic actions of the different cellular components for the survival and evolution of the cell. This mechanism is under the aegis of the Schrödinger equation that describes the changes over time of a physical system (the cell) [26]. The remaining membrane is semi-permeable, permitting an oxidative phosphorylation that is the process in which ATP is formed or chemiosmosis: entropy and chemiosmosis are complementary, and they can be considered fractal reiteration within the evolution [40]. Palpation deforms tissues and cells: we can assume that the palpatory act is the beginning of a therapeutic act on patients, where the palpated tissues gather information about the operator (awareness of the fascial system). In biological systems, it is the ability to maintain cellular homeostasis through the evaluation of information and the energy transfer resulting from a cell-cell interaction [40]. Cell-cell junctions are specialized membrane areas consisting of multiprotein complexes that provide contact between the neighbouring cells or between a cell and the extracellular matrix. These intercellular contacts may be transient, permitting the passage of information and mesenchymal cells, or they can create stable bonds to form a barrier [41]. Types of specific transmembrane junctions are defined depending on the specific receptor, which mediates the transmission of information between the membrane and the cytoskeleton; furthermore, receptors are regulated by membrane trafficking, the existence of membrane lipids and the membrane shape when a mechanical stress occurs. These junctions rapidly communicate membrane deformation to other cells, irrespective of the liquids present around such as the extracellular matrix [41]. Cellular aggregation through intercellular junctions forms a moving entity as a viscous fluid with irregular behaviour (entropic), with the aim to minimize friction across cell surfaces [42]. Cell aggregates are always present in the extracellular matrix, interstitial fluids and the bloodstream and lymphatic flow; living cells behave like fluid-filled sponges. Taking a "liquid" behaviour, cellular movement within these liquids creates waves or vibrations, which results in another way of communication between cells and tissues: vibration frequency is measurable with a spatial and temporal scale [43]. This fascial "wet network" strengthens our RAIN theoretical model [8].

Cytoskeleton

The cytoskeleton is a complex network of interlinking of microtubules and cytoplasmic filaments. The cytoskeleton is a structure that helps cells maintain their shape and internal organization and provides specific characteristics to them (such as stiffness, flexibility and motility). Many forces acting on a junction between cells come from within, especially when a junction is combined with the actomyosin complex that forms within the cytoskeleton (intrinsic force), via the cadherin-catenin complex [41]. This connection allows the cell to communicate faster, especially if the extracellular matrix (biopolymers in a three-dimensional context) is in small quantities; the same transfer of information (mechanometabolic) using the same modalities could spend hours to arrive at other tissues; this behavioural and temporal context of the cell is not completely understood [41]. Cell deformation by intrinsic forces could give rise to very fast or extremely slow messages. We can assume that palpation can create mechanical stresses that continue over time. Cells are deformed following vectors, as the shape of the man's footprinted in the sand [41]. Cellular morphology affects the extracellular matrix shape, influencing how the mechanometabolic resulting message will be transported: slow, fast or conditioning its direction [44]. Probably this kind of "mirror" behaviour would allow the cell to better respond to stress solicitation, improving its adaptation [45]. The cytoskeleton plays an important role for cell conformational memory, thanks to a metabolic regulator as the target of rapamycin (TOR), which acts on polymerization of actin, determining its cytoskeletal conformation [45]. A fundamental role played by the actomyosin complex is collecting information outside the cell and at the same time reinforcing the cell. The actin forms a network able to branch out within the cell by G-actin monomers exchange; these monomers place themselves in the terminal part of the neighbouring filament [11]. The growth of the actin filament pushes against the inner cell membrane, creating small curvatures on the outer surface of the plasma membrane (lamellipodium) or longer ramifications (filopodium): this phenomenon is called treadmilling of actin [11]. The growth of these ramifications is interrupted when capping proteins will be associated at the terminal part of the actin filament (F-actin); instead, their disassembly is due to the action of depolymerizing factors (actin depolymerizing factor - ADF) [11]. The treadmilling of the actin phenomenon is entropic. This behaviour allows the cell to be aware of its surroundings; the cell changes its morphology or implements specific mechanisms of mechanotransduction by the resistance encountered. The myosin acts as a stabilizing protein, located ventrally and dorsally each branch; it produces a contractile force equal to the tension produced by the actin filament. Myosin counterbalances and simultaneously sends back the mechanical information into the cell [46]. These two opposing forces make the membrane stiffer: the actin that pushes out and the myosin that pulls inward. This is not a negative result, tight as a guitar string; the cell becomes more sensitive to the changes in tension and improves the accuracy of mechanotransduction [46]. The information received readily crosses the cell nucleus (microseconds) and produces an instantaneous response of the genes [47].

Microtubules

The cell must have an entropic organization because it is aware that the knowledge is asymptotic, which means that the stimuli received come from an ambiguous environment and the cell can change its morphology in real time because of this absence of syntropy. The fact that it is impossible to know the cellular external environment is in accordance with Heisenberg's uncertainty principle in quantum mechanics: it asserts a fundamental limit to the precision with which certain physical properties of a particle, such as position and momentum, can be measured (at the same time or in subsequent occasions) and known [48]. Microtubules (MTs) form a part of the cytoskeleton that provides cells with structure and shape, or more specifically, microtubule-associated proteins (MAPs) [5]. MTs are tubular polymers of tubulin, formed by the polymerization of a dimer of two tubulin proteins that might have a different length. Each tubulin is defined as a dimer (two monomers: alpha and beta), with a dipole (positive and negative electrical charge); the latter gives the electromagnetic property to the tubulin structure and the MAPs complex [5]. Motor proteins (dynein and kinesin) are found in all MAPs, and they can move rapidly along the MPs, transporting different molecules [5]. The same motor proteins can help MPs in contraction, to improve cell adaptation resulting from a mechanical information received; MPs can produce forces measurable in pN (piconewton) [47]. MAPs are a more stable network than the actomyosin filaments, keeping the memory of mechanometabolic events longer and encoding them faster. According to Sherrington, MAPs can be compared to a cell of the nervous system put inside another cell [5]. MAPs carry electromagnetic information and vibrations as a rapid communication tool, in response to a cellular morphological change, towards the inside of the cell (DNA) and towards the neighbouring cells. This mechanism can be compared to a cellular awareness [5]. Electromagnetism is associated with another law of quantum physics, the "non-local entanglement": when two cells or molecules are in contact, this connection creates an unbroken microbiological link; in this way, every cell is aware of what happens to another cell, no matter how far away [5]. During a morphological change, the energy released by MAPs and by other cellular structures is minimal or quantum. It is possible to summarize this quantum energy by the formula: E = hv ("E" represents a particle's energy, "v" is the oscillation, "h" is the Planck's constant) [5]. Another example of the close relationship between biology and quantum physics is represented by biophotons or quantum particles. These are contained and then emitted by the entire fascial system (ultraweak photon emission or UPE), both liquid and solid, as already discussed in another article [49]. DNA carries and produces electromagnetic fields and UPE; its filaments carry electrons, produced internally or in other cellular structures, or even from other distant cells. The DNA adapts itself to cellular morphological changes, increasing the transcription of genes activated by specific regions of DNA which are sensitive to the flow of electromagnetic energy: electromagnetic response elements or EMRE [39]. The deformation of the cellular structures also activates the transcription of other genes, which are specific to a mechanical stimulus [50]. Palpation is an instrument useful for interacting with cells, not only locally but also in a long-distance, thanks to the different means of communication provided by the tissue on the surface and below. Further scientific and experimental research will be needed to better understand how the principles of quantum physics work within biology. We quote a sentence that summarizes our intent to associate these two themes, the fascial system and the palpation: “quantum physics and electro-dynamics shape all molecules and thus determine molecular recognition, the workings of proteins, and DNA…all this is quantum physics and a natural basis for life and everything we see [14].”

## Conclusions

The fascial system supports, protects, evolves and connects the human body. It can be divided into solid and liquid fascia, closely inter-linked, without interruption between the different components, making the subdivision of the fascia into layers unnecessary. Healthcare professionals, such as medical doctors and physiotherapists, have different clinical tools for patient assessment, including palpation. The touch meets the skin as the first fascial tissue, but the resulting cell deformation can get deeper and it can reach the DNA of different cell tissues. The morphological deformation of the cellular components starts numerous mechanometabolic and electromagnetic messages; this information will affect the entire body structure, as the palpated area and the remaining non-palpated tissues. The mechanisms that allow cells to communicate with each other are based on the principles of physiology and quantum physics. The article reviewed these scientific concepts to understand the importance of palpation in the clinical setting and the complexity of cellular behaviour, not completely understood. Further research and studies are needed to implement our knowledge of two fundamental sciences: Biology and Physics.
